# Stress-related psycho-physiological disorders: randomized single blind placebo controlled naturalistic study of psychometric evaluation using a radio electric asymmetric treatment

**DOI:** 10.1186/1477-7525-9-54

**Published:** 2011-07-19

**Authors:** Salvatore Rinaldi, Vania Fontani, Lucia Aravagli, Piero Mannu, Alessandro Castagna, Matteo Lotti Margotti, Barbara Rosettani

**Affiliations:** 1 Department of Neuro-Psycho-Physio Pathology and Neuro Psycho Physical Optimization, Rinaldi Fontani Institute, Viale Belfiore 43, Florence 50144, Italy; 2 Medical School of Occupational Medicine, University of Florence, Largo Piero Palagi, Florence, 50139, Italy

## Abstract

**Background:**

The aim of this study is to investigate the effects of a radio electric asymmetric treatment on psycho-physiological disorders (PPD). PPD are often stress related and are under the unconscious control of the patient and cannot be traced back to any serious physical disease. The brain stimulation treatment protocol used is called Neuro Psycho Physical Optimization (NPPO) with a Radio Electric Asymmetric Conveyer (REAC) device.

**Methods:**

Psychological stress and PPD were measured for a group of 888 subjects using the Psychological Stress Measure (PSM) test, a self-administered questionnaire. Data were collected immediately before and after the 4-weeks of REAC treatment cycle.

**Results:**

This study showed a significant reduction in scores measuring subjective perceptions of stress for subjects treated with a cycle of NPPO REAC treatment. At the end-point the number of subjects reporting symptoms of stress-related PPD on the PSM test was significantly reduced, whereas in the placebo group the difference was not significant.

**Conclusion:**

A cycle of NPPO treatment with REAC was shown to reduce subjective perceptions of stress measured by the PSM test and in particular on PPD.

**Trial Registration:**

This trial has been registered in the Australian New Zealand Clinical Trials Registry (ANZCTR) with the number: ACTRN12607000463471.

## Background

A high proportion of patients with general distress were suffering only from PPD (also known as "Psychosomatic disorders" or "maladaptive illness") classified as "psychological factors affecting medical condition" in the Diagnostic and Statistical Manual of Mental Disorders (DSM-IV-TR, APA, 2000) (code 777). PPD are characterized by psychological and physical symptoms that are caused by stress and emotional factors and involve one o more systems.

The physiological changes involved are those that normally are related with specific emotional states, but in PPD these changes are more intense and sustained.

Common PPD include insomnia, temporal-mandibular joint problems (in particular joint pain), migraine headaches, tension headaches, attention deficit and hyperactivity disorder (ADHD), arthritis, functional diarrhea, ulcerative colitis, essential hypertension, asthma and primary dysmenorrhoea. These symptoms are often medically unexplained [1,2] and often difficult to treat. The purpose of this work is to verify if the use of NPPO REAC treatment was effective in reducing subjective perceptions of stress as measured by a validated questionnaire, the PSM test [3,4]. This questionnaire allows to classify the stress-level and the stress-related symptoms of PPD [5] of the subjects.

## Methods and Materials

The study was performed in accordance with the Declaration of Helsinki, with the Società di Ottimizzazione Neuro Psico Fisica e CRM Terapia's institutional ethics committee approval, and all subjects provided written informed consent.

### Participants

888 subjects were included in the study from an initial group of 1453 of patients who attended the Rinaldi-Fontani Institute in Florence, Italy (*Additional file *1). These patients showed different types of stress-related PPD such as tension and migraine headaches, essential hypertension, anxious tremors, colitis, irritable bowel syndrome, bruxism, neck and back pain, chronic pain syndrome, bronchial asthma, peptic ulcer disease, skin disorders and insomnia. Subjects were not taking any psychotropic medication.

### Sample size and randomization

This is a naturalistic study therefore patients, with any type of stress-related pathology, came spontaneously to our medical centre and were observed in the normal clinical practice.

In order to have a group of control for comparing the results from the treated patients, two groups were created. Therefore, subjects were randomly selected for each group with a simple computerized randomization by an external operator. and were divided into 2 groups: Group A subjects receiving active treatment and Group B subjects receiving placebo treatment in a specific room.

Even if this study took place in Italy with Italian investigators, we could not find an Italian registry in the World Health Organization - International Clinical Trials Registry Platform http://www.who.int/ictrp/network/primary/en/index.html. Therefore we decided to select the first in the list that was the Australian New Zealand Clinical Trial Registry (ANZCTR) and registered (n° ACTRN12607000463471).

### Demographic characteristics

Group A included 688 subjects: 401 (58,28%) females, average age 42.3 ± 11.3 yrs, and 287 (41,71%) males, average age 41.1 ± 11.4 yrs. These patients were treated with a cycle of NPPO of "active" REAC. Group B included 200 subjects: 123(61,5%) females, average age 48.8 ± 19.4 yrs, and 77 (38,5%) males, average age 45.8 ± 18.5 yrs. These patients were treated with a cycle of NPPO of "Placebo" REAC. Table 1.

**Table 1:** Demographic characteristics

	Group A	Group B	Total
**Sex**			
Female	401 (58,28%)	123 (62%)	688 (100%) (A)
Male	287 (41,71%)	77 (%39)	200 (100%) (B)

**Age**			
N	688	200	888
Mean	41,8	47,7	43,13
SD	11,35	19,14	13,71

Table legend text. Demographic characteristics.

### Psychological test and psychiatric assessment

The Psychological Stress Measure (PSM) [3,4,6] was specifically developed to detect the stress levels in non-clinical population. The PSM is usually a 49 items self-report paper and pencil questionnaire; but in this study we used an electronic version to collect and process the data, and to analyze the results. Each item is based on clusters of stress conditions: loss of self-control, irritability, psycho-physiological disorders, confusion, anxiety, depression, physical pain, hyperactivity and agitation. Patients were asked to answer the questions about their psychological stress using a 4-points scale to describe the intensity of their condition (very much = 4, much = 3, little = 2, none = 1). The final score is expressed in Total Points (TP). Furthermore, in order to detect the presence of the PPD symptoms, scores obtained from items hearing voices that other people do not hear (16), feeling afraid to go out of your house alone (25), your feelings being easily hurt (34), nausea or upset stomach (40) were specifically used. Both groups of subjects were clinically evaluated at t0 (before treatment) and t1 (after treatment) by a psychiatrist. At baseline and in the total of the sample, the mainly psychiatric diagnosis (according with DSMIV-TR) have been "subthreshold" forms of Generalized Anxiety Disorder (N = 557 patients) and Anxiety Disorder Not Otherwise Specified (NOS) (N = 114). After the treatment, all the symptoms of these mild clinical conditions have shown a remarkable improvement. In the remaining 217 subjects non relevant clinical aspect has been detected.

Six-hundred-eighty-eight subjects (average PSM test total scores 122.53 ± 6.75) were treated with NPPO REAC therapy; 200 (average PSM test total scores 122.96 ± 7.041) were treated with "placebo NPPO REAC" and used as control.

### Description of the REAC and of the brain stimulation treatment protocol NPPO

The REAC is an innovative medical device [7,8] aimed to promote a reduction of the dysfunctional modifications in the Nervous System induced by stress and psychological factors, as reported by several medical articles [9-20]. The REAC brain stimulation protocol treatment consists in a cycle of 18 NPPO sessions, applied in specific points of the auricular pavilion [10]. The REAC treatment is simple, rapid, non-invasive and applied without pain. The instrument that we used is registered under the trademark Convogliatore di Radianza Modulante - CRM by ASMED, Italy.

### Statistical analysis

Statistical analysis was performed using the Number Needed to Treat Analysis (NNT) Table 2. For comparing total points before and after the treatment (placebo), the Wilcoxon Signed Rank Test was used (*Additional file *2*and *3) while the McNemar (*Additional file *4*and *5) test analyzed the presence of the PPD symptoms. Test, and all results P < 0.05 has been considered statistically significant.

**Table 2:** NNT analysis

	Present	Absent	Total
**Given (Group A)**	138	550	688
**Not Given (Group B)**	148	52	200
**Total**	286	602	888
			
**Risk of Outcome in Treated Group:**		0,2000	
**Risk of Outcome in Control Group:**		0,7400	
**Absolute Risk Reduction:**		0,5400	
**Relative Risk:**		0,2700	
**Relative Risk Reduction**		0,7300	

**Number Needed To Treat**		**1,8500**	

Table legend text. Number Needed to Treat Analysis.

## Results

Before the "active" treatment among 688 subjects, 512 (74.41%) were positive for stress-related psycho physiological disorder (PPD), and in the 200 patients of the "placebo" group these kind of disorders were detected in 150 (75%) subjects.

After "active" REAC-treatment, only 138 of 512 patients (26.9%) still presented symptoms of stress-related PPD (McNemar Test Chi-Square = 372,003, Asymp. Sig. = 0,000). In terms of percentage, migraine headache and bronchial asthma still remains in about 65% and 30% of these "poor-responder" subjects, respectively, whereas in the "placebo" group, a wider spectrum of symptoms (mainly migraine headaches, essential hypertension, anxious tremors, neck and back pain, chronic pain, insomnia, etc) were observed in 148 of 150 subjects (98.6%).

**Figure 1:**
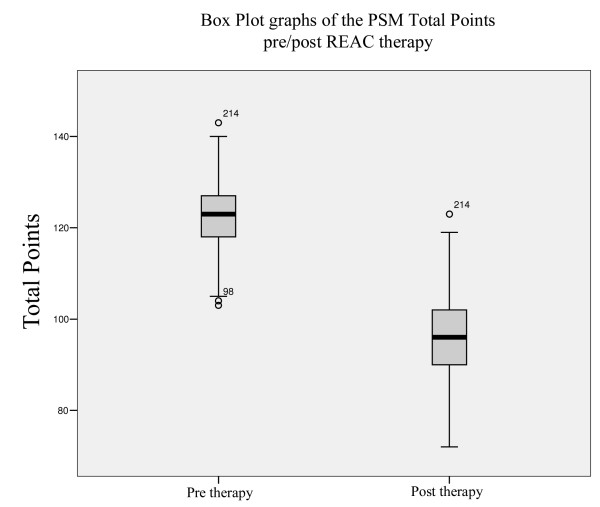
**Box Plot Graphs (treated)**. Box Plot Graphs of the PSM total points of the real treatment.

**Figure 2:**
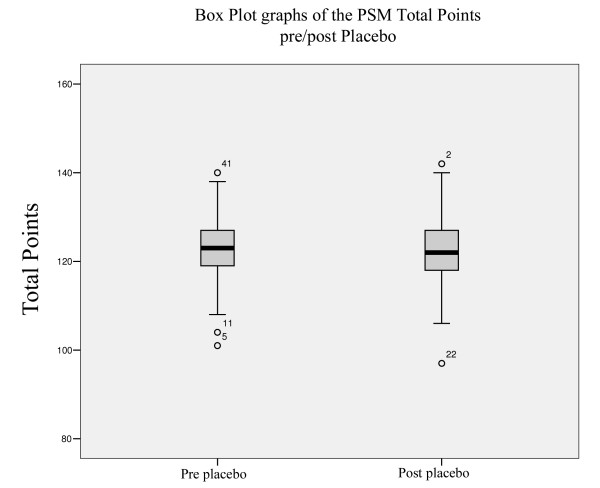
**Box Plot Graphs (placebo)**. Box Plot Graphs of the PSM total points of the placebo.

In Group A, the TP scores decreased significantly from 122.3 to 96.01, Figure 1 (Wilcoxon Signed Rank Test Z = -22,735, Asymp. Sig (2-tailed) = 0,000); in Group B, the decrease of TP scores from 122.96 to 122.11, was not statistically significant (Wilcoxon Signed Rank Test Z=-0,914, Asymp. Sig (2-tailed) = 0,361), Figure 2, Table 3.

**Table 3:** PSM test results

Subjects	Total points PSM Test	PPD n. of subjects (%)	Wilcoxon Test
**Group A (n. 688) (t0)**	122.53 ± 6.747	n. 512	Asymp. Sig. (2-tailed) 0.000
**Group A** **(t1)**	96.01 ± 8.520	n. 138 (20% of 512)	Z=-22.735 P < 0.005

**Group B (n. 200) (t0)**	122.96 ± 7.041	n. 150	Asymp. Sig. (2-tailed) 0.361
**Group B** **(t1)**	122.11 ± 7.450	n. 148 (98.6% of 150)	Z=-0.914 P > 0.005

Table legend text. PSM test results: Total points and Wilcoxon test results obtained in group A and in group B, before (t0) and after (t1) NPPO REAC treatment/placebo. Values of Total points are expressed by means ± Standard Deviation. *P < 0.05.

There is no significant correlation between the results of the PSM total points before/after Therapy and the age or the gender (*Additional file *6).

## Discussion

This study is the evolution in longitudinal terms of a previous study, and it is specifically targeted to assess each one stress-related clusters of PSM test.

Recent literature data showed that the medical use of electricity, in many kinds of applications, is probably one of the most important alternative to the "typical" psychopharmacological strategies in the management of Mental Disorders, included the stress-related PPD [21].

As pointed out Bruce S. McEwen, the brain is the key organ of the adaptive and maladaptive responses to stress [22] because it determines what is threatening and, therefore, potentially stressful, as well as initiating the behavioral, as well as many of the physiological responses to the stressors, which can be either adaptive or damaging [23-25]. All the adaptations are also expressed by changes of brain electrical activity. All neurological functions are governed mainly by two neurotransmitters: the excitatory glutamate and inhibitory gamma amino butyric acid - (GABA). Their release depends on the electrical changes due to ion flows. The REAC, with its exclusive high technology, reshapes these flows. This induces a progressive, stable and successful reorganization of the bioelectric activity of the central nervous system and consequently of the alterated systems and functions due to PPD in our body. We call this reorganization Neuro Psycho Physical Optimization [9-11,13-20].

## Conclusions

This research highlights the efficacy of NPPO treatment with REAC on PPD cluster of PSM test in non psychiatric subjects. These conditions represent the most comprehensive mental suffering in the general population. Further studies are needed to verify the stability over time when using more than one cycle, although it could be very difficult to obtain and to maintain a selected group, especially after treatment. Our results showed that NPPO treatment with REAC will help to speed up the physiological capability of the organism recovery, optimising the adaptive response to environmental stressors and contributing to the elimination of dysfunctional adaptive responses like PPD.

Clearly, our research presents several limitations. First, the wide variation and polymorphism of the stress-related symptomatology need to be considered in the global evaluation of our results. Second, it should be considered that REAC treatment is an innovative treatment and these patients could have had great expectations. Third, its administration is almost imperceptible and this characteristic can be the basis of the very little placebo effect. Fourth, the different mean age between the Group A and Group B could somehow conditioning the obtained results: therefore, there will be more reliable results comparing similar groups of age. Finally, a longer period of observation and the administration of further REAC-therapy cycles are necessary to assess the stability of our goals over time.

## Competing interests

Salvatore Rinaldi and Vania Fontani are the inventors of the Radio Electric Asymmetric Conveyer.

## Authors' contributions

SR and VF conceived of the study, participated in its design and coordination and in drafting of the manuscript. PM, psychiatrist, for the psychiatric clinical evaluation, LA and AC have critically revised the manuscript, MLM has done data analysis and BR professional statistician, has overseen the data analysis. All authors read and approved the final manuscript.

## Supplementary Material

Additional file 1 **CONSORT Checklist**. Consort checklist. Click here for file

Additional file 2 **Statistic of Real therapy 688**. Statistic of Real therapy 688 (Group A) Click here for file

Additional file 3 **Statistic of Placebo control 200**. Statistic of Placebo control 200 (Group B) Click here for file

Additional file 4 **Statistic of Real therapy 688**. McNemar Test of Real Therapy (Group A) Click here for file

Additional file 5 **Statistic of Placebo control 200**. McNemar Test of Placebo control (Group B) Click here for file

Additional file 6 **Statistic of Real therapy 688**. Correlation between PSM Total points and subgroups (age and gender) Click here for file
