# Supplementary material for: Stress-related psycho-physiological disorders: randomized single blind placebo controlled naturalistic study of psychometric evaluation using a radio electric asymmetric treatment
Source: Health Qual Life Outcomes. 2011 Jul 19;9:54. doi: 10.1186/1477-7525-9-54 (PMC3150240; doi:10.1186/1477-7525-9-54)
Supplement: Additional file 1 — CONSORT Checklist. Consort checklist. [file 1477-7525-9-54-S1.PDF]

CONSORT **Checklist**

PAPER SECTION And topic	Item	Description
TITLE & ABSTRACT	1	Neuro Psycho Physical Optimization with Conveyer of Modulating Radiance (CMR) in patients with stress-related psycho-physiological disorders. A randomised controlled trial to evaluate the effectiveness.<br><br>688 subjects were selected randomly from a 888 group to be subjected to a cycle of Neuro Psycho Physical Optimization (NPPO) with CRM therapy and 200 subjects were used for the control group
INTRODUCTION Background	2	It has been shown that chronic stress causes a progressive malfunction in some structures of the nervous system which contribute to generating the symptoms of stress correlated psycho-physiological disorders. In this study we have chosen a test used at an international level and validated in Italian (PSM Test) to prove that the use of an innovative instrument Conveyer of Modulating Radiance (CRM) - is effective) in reducing the subjective perception of stress, stress-related psycho-physiological disorders
METHODS Participants	3	888 subjects were included in the study from a initial group of 1453 of patients, with any type of stress-related pathology who were visiting our medical centre, specialized in Neuro Psycho Physical Optimization, working in cooperation whit the University of Firenze - Faculty of Medicine and Surgery - Master of II° Level - Neuro Psycho Physical Optimization and CRM Treatment.<br>Subjects taking psychotropic medication or suffering from psychiatric pathologies were excluded.<br>All of the subjects signed their consent for the study.<br>688 subjects were selected randomly from this group to be subjected to a cycle of Neuro Psycho Physical Optimization (NPPO) with CRM therapy and 200 subjects were used for the control group.
Interventions	4	Treatment devices.<br>The instrument used in this study is a novel biomedical radiofrequency instrument, called Radioelectric Asymmetric Conveyer (RAC) brand name Conveyer of Modulating Radiance (CRM). The empiric protocol used, called Neuro Psycho Physical Optimization (NPPO), involves the application of a probe of the CRM on specific points of the auricular pavilion for an activation time of roughly 500 mms. The NPPO protocol is activated in a precise sequence on seven auricular points. The protocol envisages 18 sessions of NPPO treatment on alternating days.<br>The group of 688 subjects treated with CRM therapy was called group A, while the control group of 200 subjects treated with an inactivated CRM was called group B**.**
Objectives	5	The purpose of the present work is to verify whether the use of CRM is effective in reducing the subjective perception of stress and the symptoms stress-related psycho-physiological disorders evaluated with the Psychological Stress Measure test (PSM).
Sample size	7	All the patients, with any type of stress-related pathology who were visiting our medical centre, specialized in Neuro Psycho Physical Optimization in five years

Randomization<br><br>Sequence generation	8	Simple randomization by an external operator
Randomization<br><br>Allocation concealment	9	Allocation not blinded for the operators
Randomization<br><br>Implementation	10	The allocation sequence was generated by an external operator.<br>The enrollment of the participants was been by medical doctors.<br>The participants' assignment to the two groups has been served as an external operator.
Blinding (masking)	11	Blinded only for the patients
Statistical methods	12	The primary analysis is comparing the relative frequencies of the presence of stress-related psycho-physiological disorders in the cluster of PSM test at baseline and at the end of the treatment with the statistical analysis test of McNemar.<br><br>An additional analysis was performed for comparing the distributions of the total scores of PSM at baseline and at the end of the treatment with statistical analysis test of Wilcoxon.

RESULTS<br>Participant flow	13	**Flowchart**<br>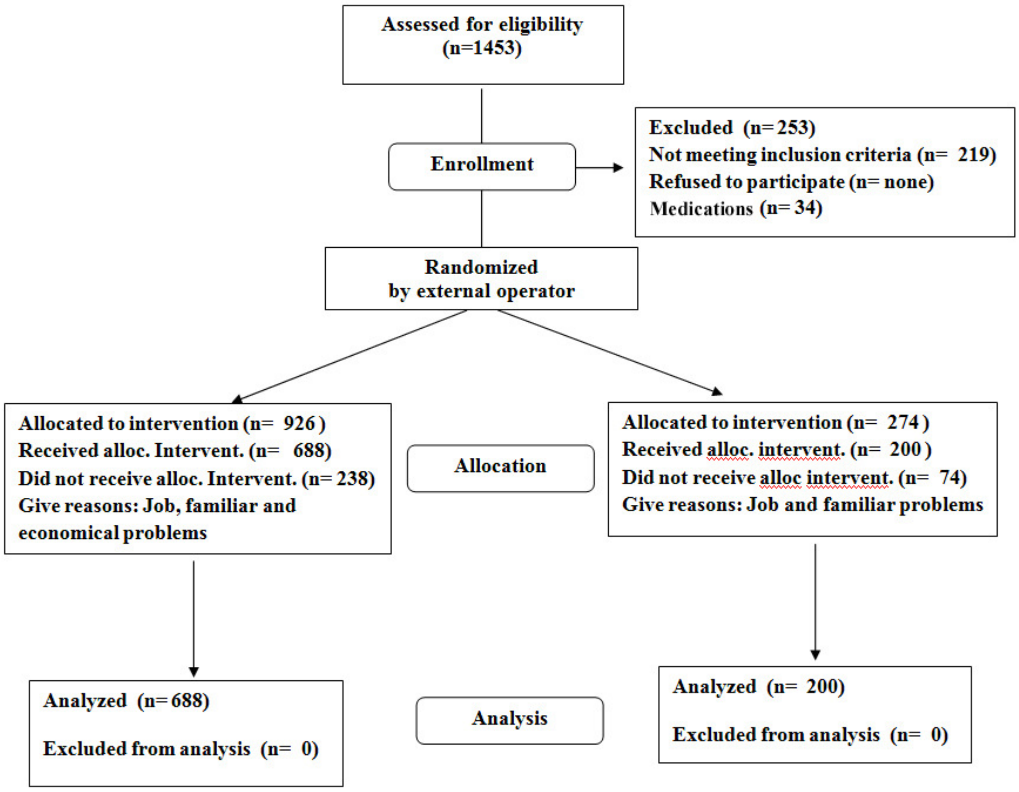

Recruitment	14	The recruitment of the patients has lasted five years:
Baseline data	15	(see table below)
Numbers analyzed	16	Group A Treated 688 / 200 Group B Placebo

**Characteristic**<br>Adult patients, males and females, with any type of stress-related disorders	**Group A Treated group**<br>(n = 688)	**Group B Placebo group**<br>(n = 200)
Males	n = 287	n = 77
Females	n = 401	n = 123
Average Males age ± SD	41.12 ± 11.38	45.80 ± 18.47
Average Females age ± SD	42.29 ± 11.30	48.88 ± 19.45
Average total scores PSM Test ± SD	122.53 ± 6.747	122.96 ± 7.041
Subjects with cluster psycho-physiological disorders at the PSM test	n = 512 (74.41%)	n = 150 (75%)

Outcomes and estimation	17	Primary Outcome: test with Psychological Stress Measure if the use of the CRM) - is effective in reducing the subjective perception of stress and psycho-physiological disorders stress-related.

Primary Outcome \ End Point

Treated (n =688)	**Pre Treatment** Subjects with psycho-physiological disorders cluster at the PSM	**Post Treatment** Subjects with psycho-physiological disorders cluster at the PSM	**McNemar test**	**P Value**
	**512 (74.41%)**	**n = 138 (20%)**	**Chi-Square = 372.003 Asymp.Sig.= .000**	**< 0.005**

Placebo (n = 200)	**Pre Placebo** Subjects with psycho-physiological disorders cluster at the PSM	**Post Placebo** Subjects with psycho-physiological disorders cluster at the PSM	**McNemar test**	**P Value**
	**150 (75%)**	**148 (74%)**	**Chi-Square = .014 Asymp.Sig.= .905**	**> 0.005**

Secondary Outcomes: Wellbeing - Neuro Psycho Physical Optimization

Secondary Outcome \ End Point

Treated (n =688)	**Pre Treatment** Average PSM Total scores	**Post Treatment** Average PSM Total scores	**Wilcoxon Test**	**P Value**
	**122.53 ± 6.747**	**96.01 ± 8.520**	**Asymp.Sig. (2-tailed) = .000 Z = -22.735**	**< 0.005**

Placebo (n = 200)	**Pre Placebo** Average PSM Total scores	**Post Placebo** Average PSM Total scores	**Wilcoxon Test**	**P Value**
	**122.96 ± 7.041**	**122.11 ± 7.450**	**Asymp.Sig. (2-tailed) = .361 Z = -0.914**	**> 0.005**

Ancillary analyses	18	Descriptives analyses were performed separately for males and females. Exploratory analyses were performed for the scores distributions.

Adverse events	19	No adverse effects.
DISCUSSION Interpretation	20	The use of electricity and magnetic fields in the biomedical studies, and in particular in the treatment of disturbances of the nervous system, is not a new idea (Ben Shachar et al., 1997; Lemyre et al., 1990; Savino et al., 2007). Nevertheless modern technology and advanced knowledge in the physical-medical field and in the neurosciences have allowed realizing the new biomedical instrument presented in this study.<br>The results suggest that a cycle of CRM therapy selectively reduces the score of the items related to anxiety depression leading to suggest a beneficial therapeutic effect of the electromagnetic field generated by the CRM. This effect can be due directly to the microwaves exciting nerve cells and/or the electric field generated by the microwaves. At present no further inference can be made and future studies will be aimed at clarifying the exact nature of the stimulus which produces the clinical effect. However, irrespective of cause that induced the change in the self-reported measure of anxiety depression of the PSM test, the results show a clinically significant improvement on the subjective perception of stress and thus an amelioration of the allostatic state, a reduction in allostatic load and a better adaptation process of the complex physiological system to physical, psychosocial and environmental challenges or stress.<br>A growing literature suggests that the symptoms of stress-related psycho-physiological disorders are the results of allostatic processes on specific cerebral areas (Bremner, 2006; Bruce & Mc Ewen, 2005; Cook & Wellman, 2004; Magarinos & McEwen, 1995; Ongur et al., 1998; Sapolsky, 1996) and our data seems to indicate that CRM is an effective instrument to optimize the responses of the CNS.<br>The obtained results showed that NPPO with CRM can reduce the subjective perception of the stress measured with the PSM psychometric test and in particular the symptoms of stress-related psycho-physiological disorders selectively in stressed individuals further suggesting a different allostatic state in the two groups.<br>This new therapy has the advantage of being painless, non-invasive and free of side-effects. Moreover the CRM therapy is a non-pharmacological and can represent an efficient support in many medical fields, because it does not interfere with the simultaneous use of other therapeutic approaches.<br>In conclusion, we propose that CRM therapy will help to speed up the physiological capability of recovery of the organism, optimising the adaptive response to environmental stressors and contributing to the elimination of dysfunctional adaptive responses.
Generalizability	21	This new therapy has the advantage of being painless, non-invasive and totally free of side-effects. The treatment is well-received by the patient and doesn't require the active collaboration of the same, acting independently of their will and being easily-accepted .<br>Another advantage is that this is not a pharmacological therapy and therefore doesn't interfere with the simultaneous use of other therapeutic approaches.
Overall evidence	22	The differences of the relative frequencies of the psycho-physiological disorders stress related between the first and the second test are significant for the patients that receive the therapy, but are not significant for the controls. We can conclude that these differences in patients that receive the therapy can be due to the effect of the therapy. Probably CRM therapy will help to speed up the physiological capacity for recovery of the organism, optimising the adaptive response to environmental stressors and contributing to the elimination of dysfunctional adaptive responses.
