# Supplementary material for: Stress-related psycho-physiological disorders: randomized single blind placebo controlled naturalistic study of psychometric evaluation using a radio electric asymmetric treatment
Source: Health Qual Life Outcomes. 2011 Jul 19;9:54. doi: 10.1186/1477-7525-9-54 (PMC3150240; doi:10.1186/1477-7525-9-54)
Supplement: Additional file 2 — Statistic of Real therapy 688. Statistic of Real therapy 688 (Group A) [file 1477-7525-9-54-S2.PDF]

Frequencies

Statistics

		Tot_Pre	Tot_Post
N	Valid	688	688
	Missing	660	660
Mean		122,53	96,01
Median		123,00	96,00
Mode		123	91
Std. Deviation		6,747	8,520
Variance		45,524	72,592
Range		40	51
Minimum		103	72
Maximum		143	123
Percentiles	10	114,00	85,00
	20	116,00	88,00
	25	118,00	90,00
	30	119,00	91,00
	40	121,00	93,00
	50	123,00	96,00
	60	124,00	98,00
	70	126,00	101,00
	75	127,00	102,00
	80	129,00	104,00
	90	131,00	107,00

Frequency Table

Tot_Pre

		Frequency	Percent	Valid Percent	Cumulative Percent
Valid	103	2	,1	,3	,3
	104	1	,1	,1	,4
	105	1	,1	,1	,6
	106	1	,1	,1	,7
	107	4	,3	,6	1,3
	108	2	,1	,3	1,6
	109	4	,3	,6	2,2
	110	3	,2	,4	2,6
	111	11	,8	1,6	4,2
	112	21	1,6	3,1	7,3
	113	14	1,0	2,0	9,3
	114	18	1,3	2,6	11,9
	115	28	2,1	4,1	16,0
	116	30	2,2	4,4	20,3
	117	29	2,2	4,2	24,6
	118	26	1,9	3,8	28,3
	119	32	2,4	4,7	33,0
	120	32	2,4	4,7	37,6
	121	38	2,8	5,5	43,2
	122	44	3,3	6,4	49,6
	123	45	3,3	6,5	56,1
	124	33	2,4	4,8	60,9
	125	42	3,1	6,1	67,0
	126	32	2,4	4,7	71,7
	127	28	2,1	4,1	75,7
	128	25	1,9	3,6	79,4
	129	37	2,7	5,4	84,7
	130	20	1,5	2,9	87,6
	131	22	1,6	3,2	90,8
	132	20	1,5	2,9	93,8
	133	10	,7	1,5	95,2
	134	9	,7	1,3	96,5
	135	4	,3	,6	97,1
	136	7	,5	1,0	98,1
	137	3	,2	,4	98,5
	138	4	,3	,6	99,1
	139	4	,3	,6	99,7
	140	1	,1	,1	99,9
	143	1	,1	,1	100,0
	Total	688	51,0	100,0	
Missing	System	660	49,0		
Total		1348	100,0		

Tot_Post

		Frequency	Percent	Valid Percent	Cumulative Percent
Valid	72	1	,1	,1	,1
	75	2	,1	,3	,4
	76	1	,1	,1	,6
	77	3	,2	,4	1,0
	78	4	,3	,6	1,6
	79	4	,3	,6	2,2
	80	4	,3	,6	2,8
	81	10	,7	1,5	4,2
	82	12	,9	1,7	6,0
	83	12	,9	1,7	7,7
	84	11	,8	1,6	9,3
	85	14	1,0	2,0	11,3
	86	22	1,6	3,2	14,5
	87	20	1,5	2,9	17,4
	88	18	1,3	2,6	20,1
	89	18	1,3	2,6	22,7
	90	25	1,9	3,6	26,3
	91	36	2,7	5,2	31,5
	92	29	2,2	4,2	35,8
	93	31	2,3	4,5	40,3
	94	27	2,0	3,9	44,2
	95	18	1,3	2,6	46,8
	96	32	2,4	4,7	51,5
	97	32	2,4	4,7	56,1
	98	34	2,5	4,9	61,0
	99	21	1,6	3,1	64,1
	100	29	2,2	4,2	68,3
	101	33	2,4	4,8	73,1
	102	20	1,5	2,9	76,0
	103	27	2,0	3,9	79,9
	104	26	1,9	3,8	83,7
	105	16	1,2	2,3	86,0
	106	20	1,5	2,9	89,0
	107	16	1,2	2,3	91,3
	108	11	,8	1,6	92,9
	109	10	,7	1,5	94,3
	110	9	,7	1,3	95,6
	111	10	,7	1,5	97,1
	112	4	,3	,6	97,7
	113	5	,4	,7	98,4
	114	3	,2	,4	98,8
	115	3	,2	,4	99,3
	116	1	,1	,1	99,4
	118	2	,1	,3	99,7
	119	1	,1	,1	99,9
	123	1	,1	,1	100,0
	Total	688	51,0	100,0	
Missing	System	660	49,0		
Total		1348	100,0		

Histogram

Tot_Pre

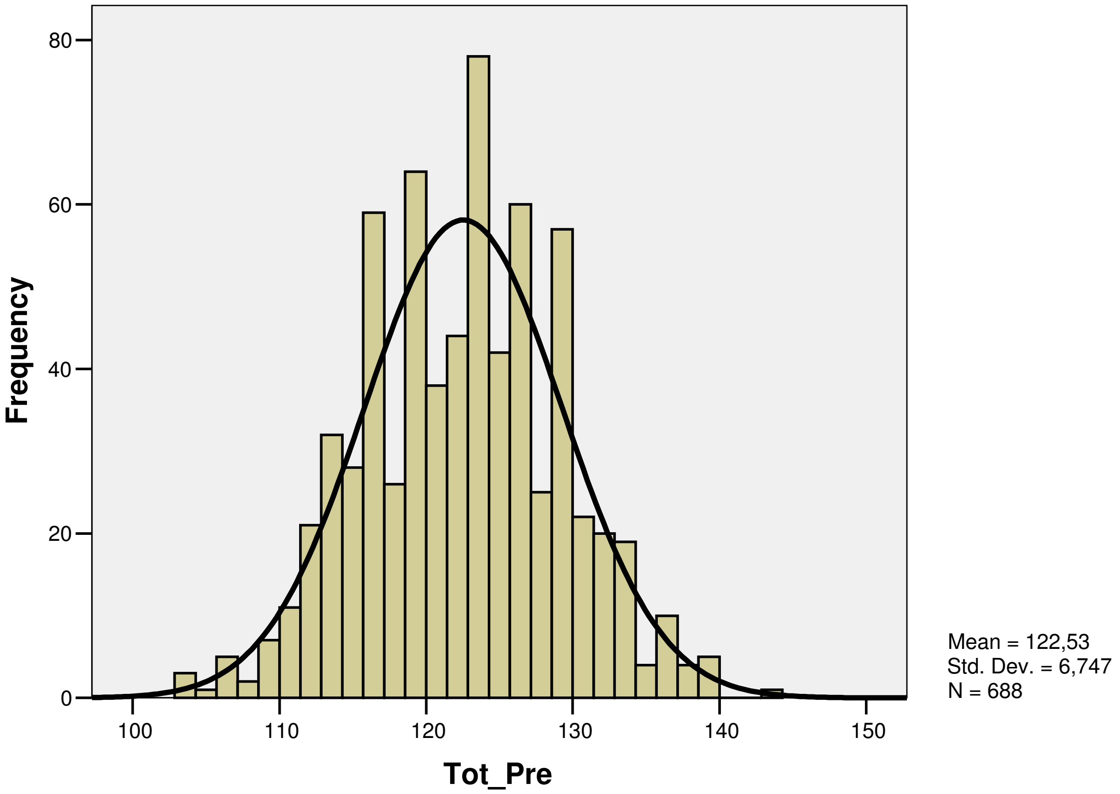

Frequency
80
60
40
20
0
100
110
120
130
140
150
Tot_Pre
Mean = 122,53
Std. Dev. = 6,747
N = 688

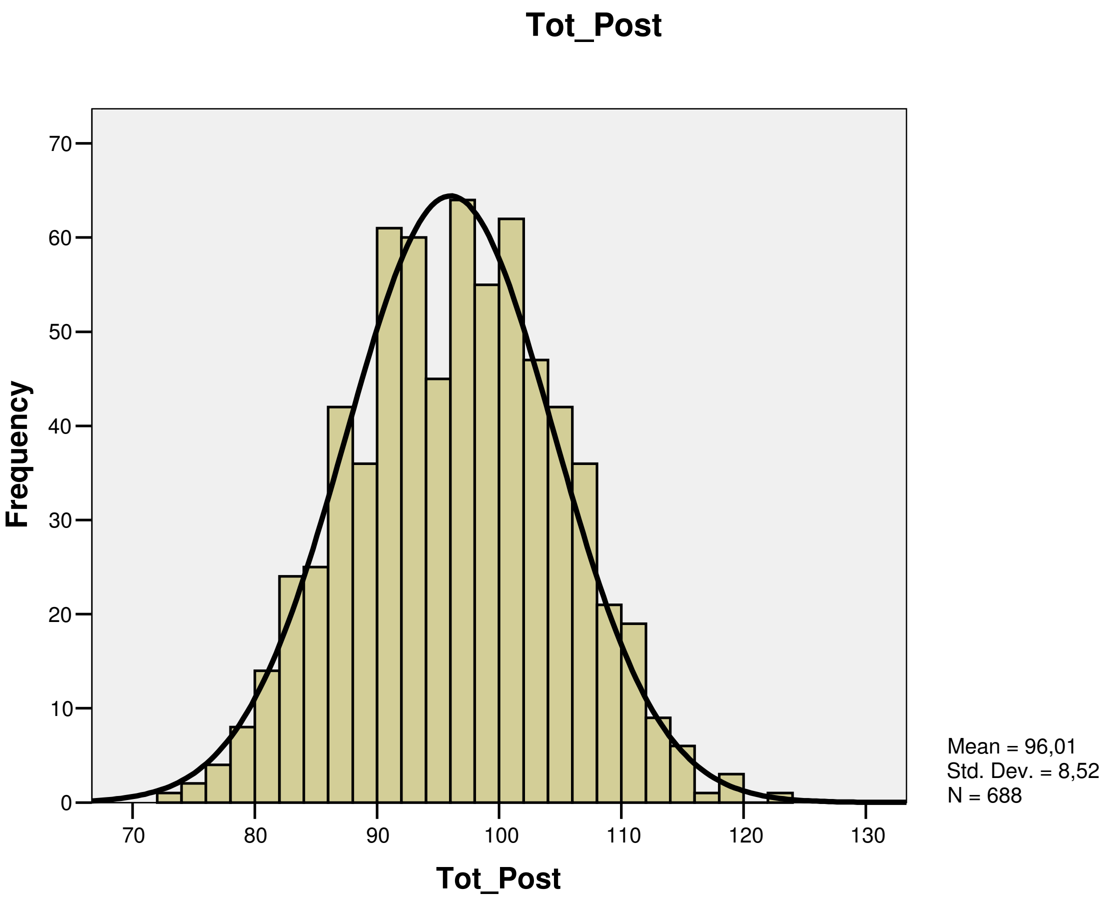

NPar Tests

Wilcoxon Signed Ranks Test

Ranks

		N	Mean Rank	Sum of Ranks
Tot_Post - Tot_Pre	Negative Ranks	688[a]	344,50	237016,00
	Positive Ranks	0[b]	,00	,00
	Ties	0[c]		
	Total	688		

a. Tot_Post < Tot_Pre

b. Tot_Post > Tot_Pre

c. Tot_Post = Tot_Pre

Test Statistics[b]

	Tot_Post - Tot_Pre
Z	-22,735[a]
Asymp. Sig. (2-tailed)	,000

a. Based on positive ranks.

b. Wilcoxon Signed Ranks Test
