# Supplementary material for: Stress-related psycho-physiological disorders: randomized single blind placebo controlled naturalistic study of psychometric evaluation using a radio electric asymmetric treatment
Source: Health Qual Life Outcomes. 2011 Jul 19;9:54. doi: 10.1186/1477-7525-9-54 (PMC3150240; doi:10.1186/1477-7525-9-54)
Supplement: Additional file 3 — Statistic of Placebo control 200. Statistic of Placebo control 200 (Group B) [file 1477-7525-9-54-S3.PDF]

Frequencies

Statistics

		Tot_Pre	Tot_Post
N	Valid	200	200
	Missing	1128	1128
Mean		122,96	122,11
Median		123,00	122,00
Mode		120	124
Std. Deviation		7,041	7,450
Variance		49,581	55,501
Minimum		101	97
Maximum		140	142
Percentiles	10	114,00	113,00
	20	116,20	116,00
	25	119,00	118,00
	30	120,00	118,30
	40	121,00	120,00
	50	123,00	122,00
	60	125,00	124,00
	70	126,00	126,00
	75	127,00	127,00
	80	128,80	128,00
	90	133,00	132,00

Frequency Table

Tot_Pre

		Frequency	Percent	Valid Percent	Cumulative Percent
Valid	101	1	,1	,5	,5
	104	1	,1	,5	1,0
	108	1	,1	,5	1,5
	110	3	,2	1,5	3,0
	111	4	,3	2,0	5,0
	112	4	,3	2,0	7,0
	113	3	,2	1,5	8,5
	114	12	,9	6,0	14,5
	115	7	,5	3,5	18,0
	116	4	,3	2,0	20,0
	117	2	,2	1,0	21,0
	118	7	,5	3,5	24,5
	119	7	,5	3,5	28,0
	120	14	1,1	7,0	35,0
	121	11	,8	5,5	40,5
	122	12	,9	6,0	46,5
	123	12	,9	6,0	52,5
	124	11	,8	5,5	58,0
	125	13	1,0	6,5	64,5
	126	12	,9	6,0	70,5
	127	10	,8	5,0	75,5
	128	9	,7	4,5	80,0
	129	7	,5	3,5	83,5
	130	6	,5	3,0	86,5
	131	2	,2	1,0	87,5
	132	4	,3	2,0	89,5
	133	3	,2	1,5	91,0
	134	6	,5	3,0	94,0
	135	6	,5	3,0	97,0
	136	1	,1	,5	97,5
	137	1	,1	,5	98,0
	138	3	,2	1,5	99,5
	140	1	,1	,5	100,0
	Total	200	15,1	100,0	
Missing	System	1128	84,9		
Total		1328	100,0		

Tot_Post

		Frequency	Percent	Valid Percent	Cumulative Percent
Valid	97	1	,1	,5	,5
	106	2	,2	1,0	1,5
	107	3	,2	1,5	3,0
	108	3	,2	1,5	4,5
	109	1	,1	,5	5,0
	110	3	,2	1,5	6,5
	111	2	,2	1,0	7,5
	112	3	,2	1,5	9,0
	113	8	,6	4,0	13,0
	114	6	,5	3,0	16,0
	115	6	,5	3,0	19,0
	116	6	,5	3,0	22,0
	117	4	,3	2,0	24,0
	118	12	,9	6,0	30,0
	119	11	,8	5,5	35,5
	120	12	,9	6,0	41,5
	121	10	,8	5,0	46,5
	122	11	,8	5,5	52,0
	123	7	,5	3,5	55,5
	124	14	1,1	7,0	62,5
	125	13	1,0	6,5	69,0
	126	8	,6	4,0	73,0
	127	9	,7	4,5	77,5
	128	7	,5	3,5	81,0
	129	7	,5	3,5	84,5
	130	3	,2	1,5	86,0
	131	6	,5	3,0	89,0
	132	4	,3	2,0	91,0
	133	7	,5	3,5	94,5
	134	3	,2	1,5	96,0
	135	2	,2	1,0	97,0
	136	1	,1	,5	97,5
	137	1	,1	,5	98,0
	138	1	,1	,5	98,5
	139	1	,1	,5	99,0
	140	1	,1	,5	99,5
	142	1	,1	,5	100,0
	Total	200	15,1	100,0	
Missing	System	1128	84,9		
Total		1328	100,0		

Histogram

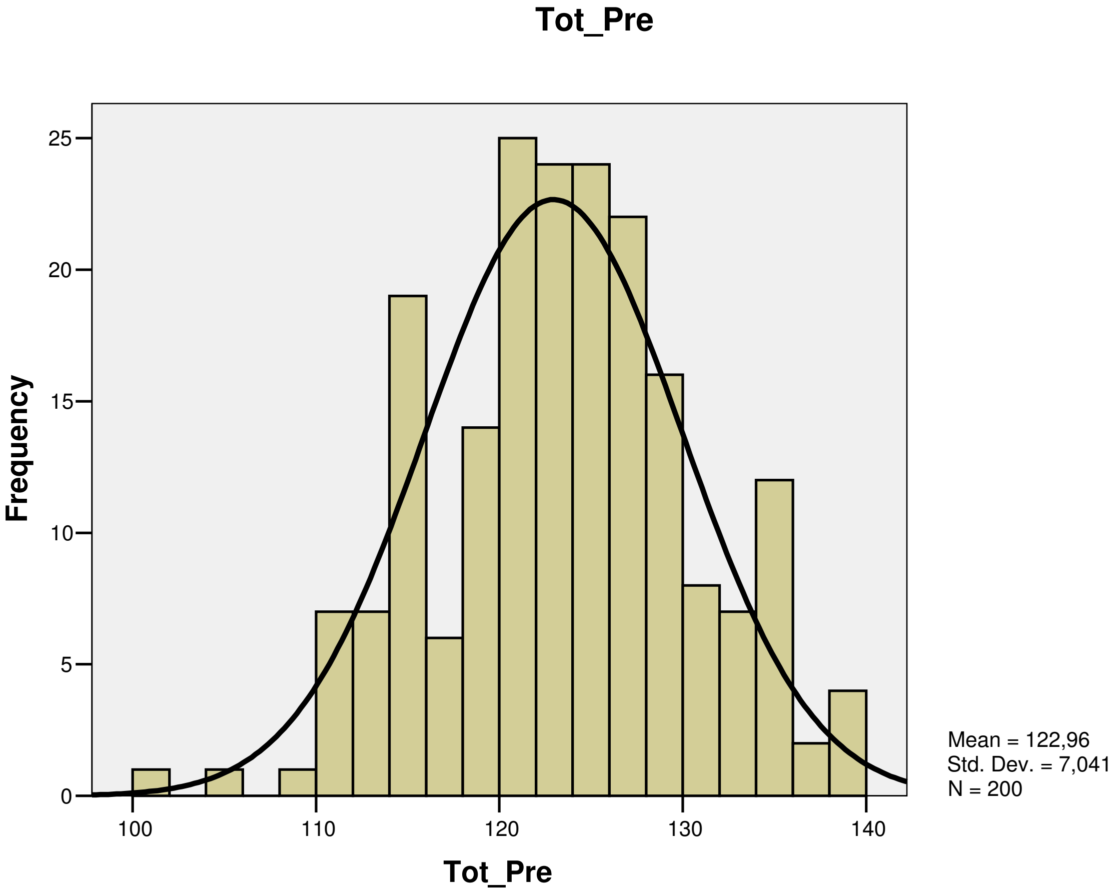
Tot_Pre
Frequency
25
20
15
10
5
0
100
110
120
130
140
Tot_Pre
Mean = 122,96
Std. Dev. = 7,041
N = 200

Tot_Post

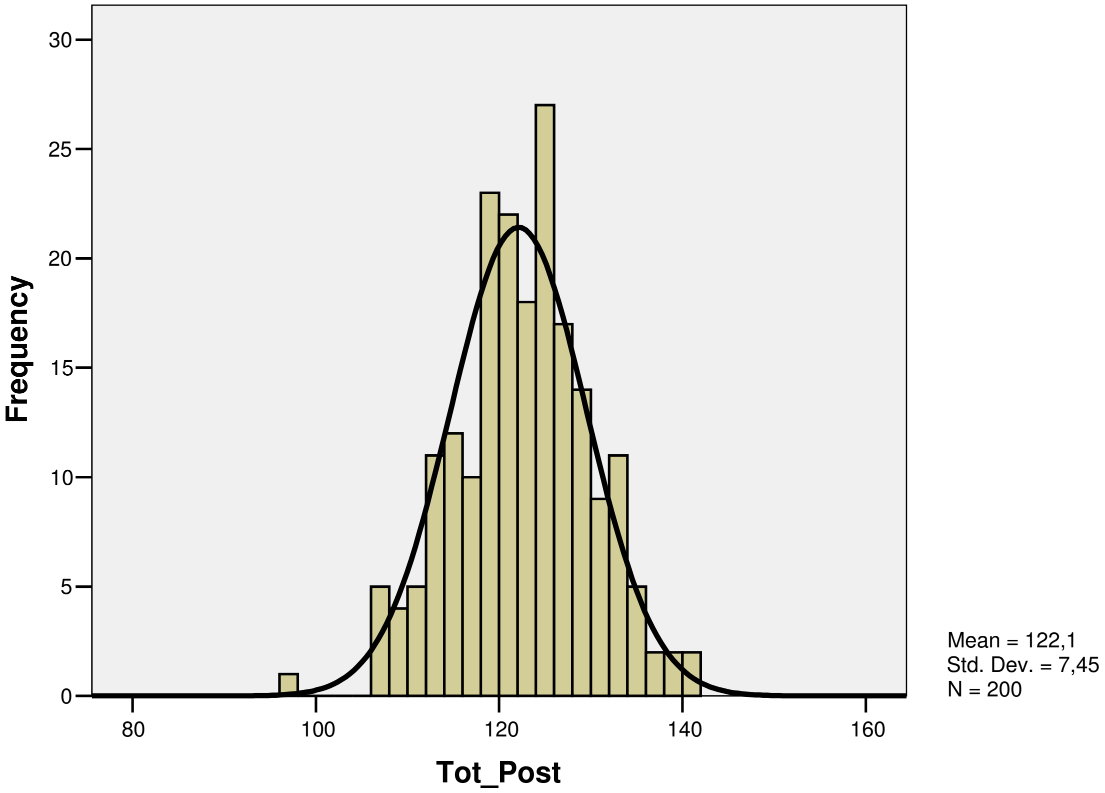

NPar Tests

Wilcoxon Signed Ranks Test

Ranks

		N	Mean Rank	Sum of Ranks
Tot_Post - Tot_Pre	Negative Ranks	96[a]	102,78	9867,00
	Positive Ranks	95[b]	89,15	8469,00
	Ties	9[c]		
	Total	200		

a. Tot_Post < Tot_Pre

b. Tot_Post > Tot_Pre

c. Tot_Post = Tot_Pre

Test Statistics[b]

	Tot_Post - Tot_Pre
Z	-,914[a]
Asymp. Sig. (2-tailed)	,361

a. Based on positive ranks.

b. Wilcoxon Signed Ranks Test
