# Supplementary material for: Stress-related psycho-physiological disorders: randomized single blind placebo controlled naturalistic study of psychometric evaluation using a radio electric asymmetric treatment
Source: Health Qual Life Outcomes. 2011 Jul 19;9:54. doi: 10.1186/1477-7525-9-54 (PMC3150240; doi:10.1186/1477-7525-9-54)
Supplement: Additional file 4 — Statistic of Real therapy 688. McNemar Test of Real Therapy (Group A) [file 1477-7525-9-54-S4.PDF]

Real therapy - McNemar Test

Crosstabs

psycho-physiological disorders

Pre treatment	Post treatment 0	Post treatment 1
0	176	0
1	374	138

Test Statistics[b]

	psycho-physiological disorders pre/post
N	688
Chi-Square[a]	372,003
Asymp. Sig.	,000

a. Continuity Corrected

b. McNemar Test
