# Supplementary material for: Stress-related psycho-physiological disorders: randomized single blind placebo controlled naturalistic study of psychometric evaluation using a radio electric asymmetric treatment
Source: Health Qual Life Outcomes. 2011 Jul 19;9:54. doi: 10.1186/1477-7525-9-54 (PMC3150240; doi:10.1186/1477-7525-9-54)
Supplement: Additional file 5 — Statistic of Placebo control 200. McNemar Test of Placebo control (Group B) [file 1477-7525-9-54-S5.PDF]

Placebo - McNemar Test

Crosstabs

psycho-physiological disorders

Pre treatment	Post treatment 0	Post treatment 1
0	16	34
1	36	114

Test Statistics[b]

	psycho-physiological disorders pre & post
N	200
Chi-Square[a]	,014
Asymp. Sig.	,905

a. Continuity Corrected

b. McNemar Test
