# Supplementary material for: Stress-related psycho-physiological disorders: randomized single blind placebo controlled naturalistic study of psychometric evaluation using a radio electric asymmetric treatment
Source: Health Qual Life Outcomes. 2011 Jul 19;9:54. doi: 10.1186/1477-7525-9-54 (PMC3150240; doi:10.1186/1477-7525-9-54)
Supplement: Additional file 6 — Statistic of Real therapy 688. Correlation between PSM Total points and subgroups (age and gender) [file 1477-7525-9-54-S6.PDF]

Correlation between The PSM Total Points (pre/post the real therapy) and Age and Gender

Correlation between Total Points and Age

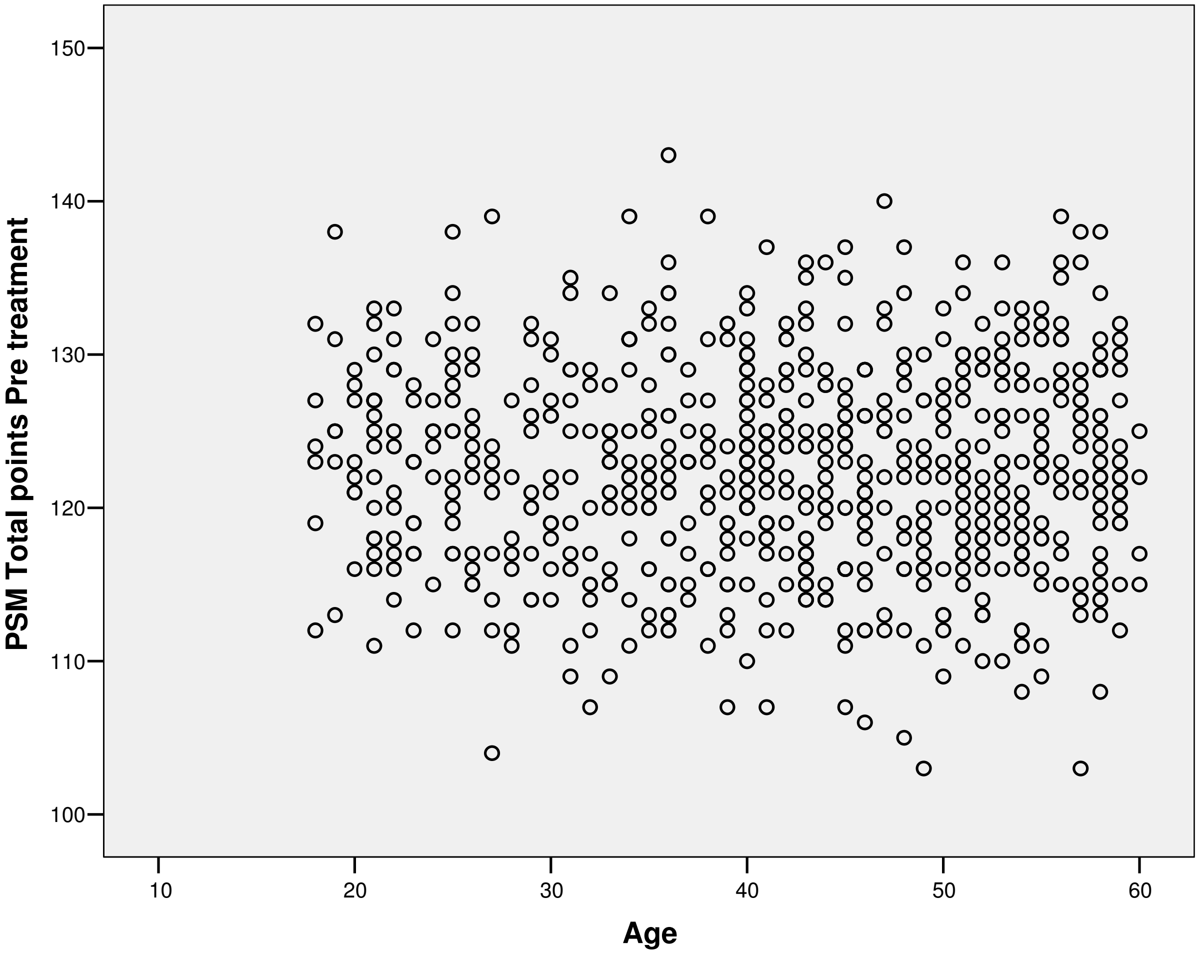

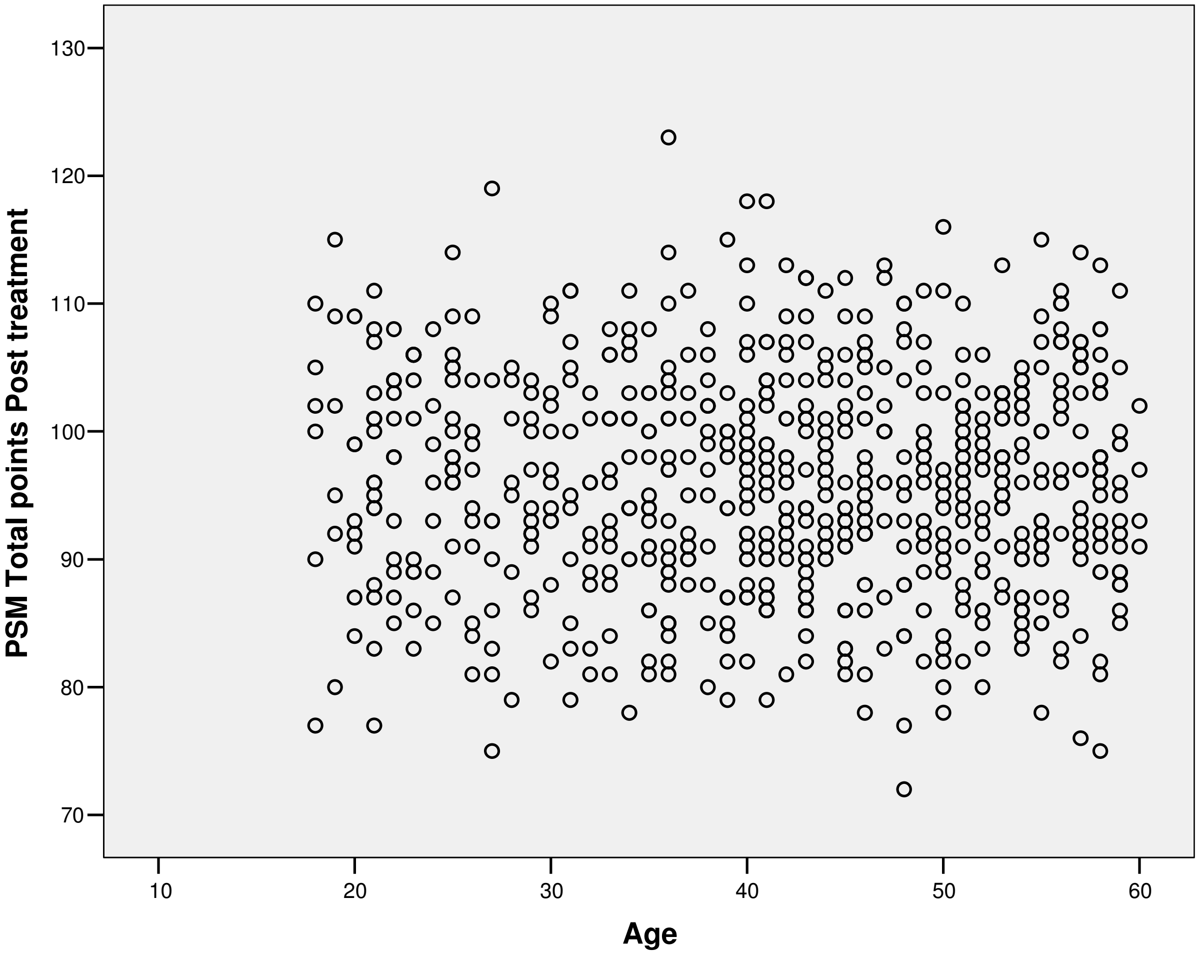

Gender:

Mann-Whitney Test: Correlation between Total Points - Pre treatment and Gender

Ranks

	Gender	N	Mean Rank	Sum of Ranks
Tot_Pre	0	401	345,42	138511,50
	1	287	343,22	98504,50
	Total	688		

Test Statistics[a]

	Tot_Pre
Mann-Whitney U	57176,500
Wilcoxon W	98504,500
Z	-,143
Asymp. Sig. (2-tailed)	,886

a. Grouping Variable: Gender

Mann-Whitney Test: Correlation between Total Points - Post treatment and Gender

Ranks

	Gender	N	Mean Rank	Sum of Ranks
Tot_Post	0	401	339,54	136157,50
	1	287	351,42	100858,50
	Total	688		

Test Statistics[a]

	Tot_Post
Mann-Whitney U	55556,500
Wilcoxon W	136157,50
Z	-,773
Asymp. Sig. (2-tailed)	,439

a. Grouping Variable: Gender
